# Clinical and Diagnostic Considerations in Laryngeal Leishmaniasis: A Systematic Review

**DOI:** 10.1002/oto2.70281

**Published:** 2026-07-30

**Authors:** Joseph Celidonio, Nicholas Hamilton, Alexandra Filipkowski, Raj Malhotra, Samantha Shave, Myat E. Mon, Kenneth Yan, Rachel Kaye

**Affiliations:** ^1^ Department of Otolaryngology–Head and Neck Surgery Rutgers New Jersey Medical School 90 Bergen St Newark 07103 New Jersey USA; ^2^ Department of Otolaryngology–Head and Neck Surgery Robert Wood Johnson/Rutgers University 675 Hoes Lane West Piscataway 08854 New Jersey USA; ^3^ Department of Pathology, Immunology, and Laboratory Medicine Rutgers New Jersey Medical School 90 Bergen St Newark 07103 New Jersey USA

**Keywords:** laryngeal, larynx, leishmania, leishmaniasis

## Abstract

**Objective:**

Laryngeal leishmaniasis is a rare, insidious manifestation of Leishmania infection with potential for life‐threatening complications. Awareness is critical for efficient and timely diagnosis. This systematic review of laryngeal leishmaniasis focuses on presentation, treatment, and the role of serological studies in the work up of these patients.

**Data Sources:**

PubMed, Scopus, and EMBASE.

**Review Methods:**

A systematic review of English literature was conducted via PubMed, Scopus, and EMBASE. Studies with both systemic and isolated laryngeal leishmaniasis data were included. Data on demographics, symptoms, diagnosis, treatment, and outcomes were extracted.

**Results:**

Of the 81 patients included, the majority were male (87.7%) and were exposed to a region endemic to Leishmania (97.3%). The mean age was 51.8 years. The most common symptoms were dysphonia (79.0%) and dysphagia (17.0%). Mean time from symptom onset to diagnosis was 18.4 months. Among patients who received serological testing for leishmaniasis, 77.2% tested positive, and those with isolated disease showed lower sensitivity. The most common treatment modalities were amphotericin (35.8%) and meglumine antimoniate (30.9%), whereas surgical treatment was pursued in only 6.7%. Most patients achieved symptom resolution (78.3%) at last follow‐up (mean 12.7 months). 12.3% of patients required a tracheostomy—time from symptom onset to diagnosis did not differ in patients with tracheostomy versus no tracheostomy (29.4 vs 16.3 months, *P* = .082).

**Conclusion:**

Laryngeal leishmaniasis is a difficult diagnosis, particularly in nonendemic regions. In fact, there is often a delay in diagnosis. Serological studies may play an important role in supporting the diagnosis.

Leishmaniasis is a systemic disease caused by the protozoan parasite Leishmania that affects 1.6 million people per year globally, primarily in endemic regions of the world.^1^ Endemic regions include Asia, Africa, the Americas, and the Mediterranean region.^2^ Nevertheless, increasing incidence in nonendemic regions has been reported in recent years, thought to be due to increasing globalization, with over 10,000 nonendemic cases reported globally from 2000 to 2021.^3^ While epidemiological data are available for cutaneous leishmaniasis (CL) and visceral leishmaniasis (VL), the incidence of laryngeal leishmaniasis (LL) is poorly defined regardless of geographic region. Considering its rarity and the potential for an insidious onset of symptoms, diagnosis of LL can be difficult. Therefore, awareness of this condition is paramount for timely clinical recognition.

The clinical presentation of LL may vary; however, initial symptoms commonly include dysphonia, dysphagia, dyspnea, odynophagia, and cough.^4^ Diagnosis of LL is often delayed due to several factors, including difficulty suspecting the disease due to its rarity.^4^, ^5^ This poses the risk for disease progression to the point of airway obstruction, making the early recognition of LL critical. This highlights the need for increased awareness of this condition, particularly in nonendemic regions where it is less frequently suspected. However, even when LL is suspected, clear diagnostic guidelines for this condition are not currently available.

There is currently no single gold standard modality for the diagnosis of leishmaniasis.^6^ Rather, a group of tests is often ordered to make an initial diagnosis of leishmaniasis and to further differentiate between individual causative species.^6^, ^7^ These tests include histological analysis of tissue samples to assess for the presence of amastigotes (Supplemental Figure S1, available online), in vitro culture for parasite isolation, detection of parasite DNA, and/or serological testing for VL.^6^ Several serological tests are available for VL, including the indirect immunofluorescent antibody test (IFAT), direct agglutination test (DAT), enzyme‐linked immunosorbent assay (ELISA), rK‐39 immunographic test (ICT), and more.^8^ Sensitivities and specificities for these tests vary, with results in endemic areas showing sensitivities of 92% or greater for rK‐39 ICT and IFAT, and specificities nearing 100% for each test.^9^ The diagnostic accuracy of these tests in nonendemic regions is not well described.

This study aims to provide a comprehensive review of peer‐reviewed literature surrounding LL. We report on patient demographics, clinical presentation, diagnostic modalities, treatment, and outcomes. Our goal is to increase awareness of the clinical presentation of LL, and highlight important considerations in the diagnostic work up of LL. Specifically, the primary outcome of this study is to identify the clinical factors that impact the requirement for tracheostomy in LL patients by comparing the time to diagnosis among patients with and without a tracheostomy. We additionally investigate the effect of immune status among LL patients on diagnostic testing accuracy, specifically focusing on the sensitivity of serological testing in patients with and without immunosuppression. An improved understanding of this disease may aid physicians in the clinical recognition and diagnosis of LL, ultimately reducing long‐term morbidity.

## METHODS

### Search Strategy

The Preferred Reporting Systems for Systematic Reviews and Meta‐Analysis (PRISMA) guidelines were followed for the inclusion of publications in this study.^10^ A search of the available peer‐reviewed literature was conducted by authors JC and NH from inception to September 16, 2024 using PubMed, Scopus and EMBASE. Search terms included (“throat OR larynx OR laryngeal AND leishmaniasis”), (“vocal cord OR glottis OR subglottis OR supraglottis AND leishmaniasis”), (“hoarseness OR dysphonia OR airway obstruction OR voice change AND leishmaniasis”). No other filters or limits were applied to the databases. This study met criteria for nonhuman subject research per the protocol of the Institutional Review Board of Rutgers New Jersey Medical School, Newark, New Jersey, and as a result, was exempt.

### Eligibility Criteria

**Figure 1:**
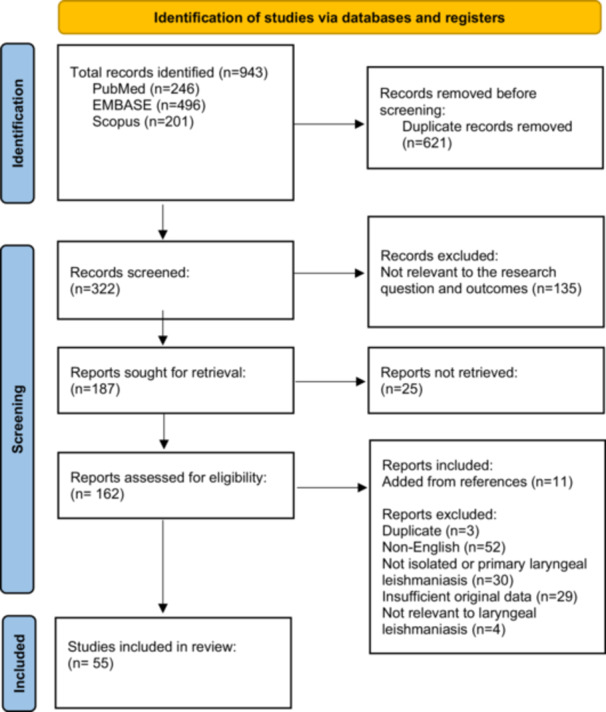
PRISMA diagram for selection of studies.

All articles were collected into the systematic review collaboration platform, Rayyan, for the facilitation of review of records.^11^ Abstract screening was then performed, followed by full text screening. All available peer‐reviewed English literature that included original data about LL was included. Exclusion criteria at each stage are described in the PRISMA flow diagram (Figure 1). All included articles were assessed using the Agency for Healthcare Research and Quality (AHRQ) bias assessment (Supplemental Table S1, available online).^12^

### Data Extraction and Analysis

The following data were extracted from each included study: Patient demographics, symptoms, time from symptom onset to diagnosis, diagnostic modalities, Leishmania species, treatment modalities, and outcomes. Inclusion criteria for the immunocompromised group were chosen to select for patients with either systemic immunosuppression and/or immunosuppression localized to the upper airway. The inclusion criteria consisted of active use of inhaled corticosteroids or systemic immunosuppressants (eg, methotrexate, corticosteroids), as well as the following conditions: diabetes mellitus, malignancy, combined variable immune deficiency, hyper‐IgM syndrome, and human immunodeficiency virus. Statistical analyses were performed using IBM SPSS v29. Univariate analyses were conducted using Chi‐squared test and Fisher's exact test, where appropriate.

## Results

### Patient Characteristics and Evaluation

A total of 81 patients with a diagnosis of LL were identified from 55 studies, with 87.7% of the overall cohort being male. Mean age was 51.8 years with a range of 17 to 88 years. The most common symptoms included dysphonia (80.2%), dysphagia (21.0%) and odynophagia (14.8%). Common physical exam findings include hepatosplenomegaly (15.5%), active cutaneous leishmania lesions or scarring from prior leishmania infection (20.7%), and lymphadenopathy (6.2%). Of the 75.3% of patients with immune status data available, 52.5% were immunocompromised. Of note, we considered localized laryngeal and systemic immunocompromise as an aggregate, in addition to separation of these groups for statistical analyses. Among the 90.1% of the cohort who had reported exposure data, 97.3% had exposure to an endemic region, which included Spain (29.6%), Italy (18.3%), Brazil (14.1%), France (11.3%), India (8.5%), Iran (2.8%), Argentina (1.4%), Ecuador (1.4%), Egypt (1.4%), Greece (1.4%), Malta (1.4%), Pakistan (1.4%), Portugal (1.4%), and Turkey (1.4%). The majority of patients had systemic involvement (69.4%) while a smaller subset had disease localized to the head and neck region (31.6%) (Table 1). Laryngoscopy findings varied greatly across studies. Descriptions were categorized as either generalized laryngeal abnormalities (33.8%) or distinct laryngeal lesion(s) (66.2%). Among those with distinct lesions, 68.9% had a single lesion while 31.1% had multiple lesions. The most common laryngoscopy findings included lesions described as granulomatous (24.6%), polypoid (22.6%), and erythematous (22.6%), however multiple different types of descriptors were used. The most commonly involved laryngeal subsite was the true vocal cords (48.2%), followed by the epiglottis (16.1%) (Table 2) (Supplemental Figure S2, available online).

**Table 1:** Demographic and Clinical Data

	Total
Subjects, N	81
Age in years, mean (range)^a^	51.8 (17‐88)
Sex, n = 81 (100%)	
Male	71 (87.7)
Female	10 (12.3)
Immunocompromised, n = 61 (75.3%)^b^	
Yes	32 (52.5)
No	29 (47.5)
Exposure to Endemic Region, n = 73 (90.1%)^c^	
Endemic	71 (97.3)
Nonendemic	2 (2.7)
Symptoms, n = 81 (100%)	
Dysphonia	65 (80.2)
Dysphagia	17 (21.0)
Odynophagia	12 (14.8)
Stridor	3 (3.7)
Dyspnea	10 (12.3)
Cough	7 (8.6)
Weight loss	7 (8.6)
Physical exam findings, n = 58 (71.6%)	
Hepatosplenomegaly	9 (15.5)
Cutaneous lesion(s) (active or scarred CL)	12 (20.7)
Lymphadenopathy	5 (6.2)
Systemic Involvement, n = 79 (97.5%)^d^	
Systemic	54 (68.4)
Localized	25 (31.6)

Number and percentage of patients with data available for each category included next to each category header.

^a^ 5 age values were estimated due to limited data availability.

^b^ 20 patients missing immunocompromise data.

^c^ 8 patients missing endemic region data.

^d^ 2 cases with unclear systemic involvement were excluded.

**Table 2:** Diagnostic Data

	Total
Symptom onset to diagnosis, months, mean (range)	18.4 (0‐120)
Laryngoscopy findings, n = 68 (84.0%)	
Generalized	23 (33.8)
Distinct lesions	45 (66.2)
Single lesion^a^	31 (68.9)
Multiple lesions^a^	14 (31.1)
Description of lesions, n = 62 (76.5%)	
Erythematous	14 (22.6)
Granulomatous	15 (24.2)
Polypoid	14 (22.6)
Leukoplakic	7 (11.3)
Nodular	4 (6.4)
Other/nonspecific	8 (12.9)
Laryngeal subsite involvement, n = 81 (100%)	
True vocal cords	39 (48.2)
False vocal cords	4 (4.9)
Epiglottis	13 (16.1)
Other	13 (16.1)
Unspecified/Generalized	21 (25.9)
Biopsy, n = 79 (97.5%)^b^	
Diagnostic	74 (93.7)
Lesional excisional	5 (6.3)
Serological studies, n = 44 (54.3%)	
Positive	34 (77.2)
Negative	10 (22.8)
Immunofluorescent antibody test, n = 19 (43.2%)	
Positive	15 (78.9)
Negative	4 (21.1)
Enzyme‐linked immunosorbent assay, n = 9 (20.5%)	
Positive	9 (100)
Negative	0
Leishmania species, n = 81 (100%)	
*L. donovani*	16 (19.8)
*L. infantum*	15 (18.5)
Unspecified	44 (54.3)
Other	6 (7.4)

Number and percentage of patients with data available for each category included next to each category header.

^a^ Only recorded for cases with distinct lesion(s).

^b^ 2 diagnoses made on autopsy.

Average time from symptom onset to diagnosis was 18.4 months. In this cohort, 97.5% of patients received a biopsy; among these patients, 74 (93.7%) had a diagnostic biopsy, 5 (6.3%) underwent a lesional excisional biopsy, and 2 (2.5%) were diagnosed on autopsy. Of the 5 excisional biopsies, 4 comprised initial tissue sampling for diagnostic purposes, and 1 represented a subsequent sampling following a previously inconclusive diagnostic biopsy. In this cohort, 54.3% of patients had serological testing done with 77.2% of these patients having positive test results. Leishmania species were most commonly unspecified (54.3%), while *L. donovani* and *L. infantum* were the two most commonly identified species (19.8% and 18.5%, respectively) (Table 2). The most common serological tests performed were IFAT (43.2%) and ELISA (20.5%), which had a sensitivity of 78.9% and 100%, respectively (Table 2). Isolated LL showed a lower sensitivity on serology compared to systemic leishmaniasis (63.6% vs 90.9%, *P* = .031) (Table 3).

**Table 3:** Serologic Sensitivity in Isolated vs. Systemic Leishmaniasis

	Systemic leishmaniasis, n (%)	Isolated laryngeal leishmaniasis, n (%)	*P*‐value
Negative serology	2 (9.1%)	8 (36.4%)	.031
Positive serology	20 (90.9%)	14 (63.6%)	

### Treatment and Outcomes

Patients were most commonly treated medically with antifungal (40.7%) and antimoniate drugs (35.8%). The most common medications administered within these drug classes were amphotericin (35.8%) and meglumine antimoniate (30.7%). Among the overall cohort, 6.3% of patients underwent surgical excision of lesions, and 12.3% required a tracheostomy with the indication being active airway obstruction in 80.0% of these patients, concern for airway obstruction in 10.0%, and oropharyngeal bleeding in 10.0%. Among the 10 patients who required a tracheostomy, 5 patients had outcome data regarding decannulation versus persistent need for a tracheostomy, and all patients with this data available were decannulated (100%). The remaining 5 patients had no specific mention of decannulation nor persistent tracheostomy requirement. Treatment outcome was available in 82.7% of patients; among these patients, 77.6% had resolution of some or all symptoms after initial treatment for leishmaniasis, while 22.4% had report of persistent or recurrent symptoms (Table 4).

**Table 4:** Treatment and Outcomes

	Total
Tracheostomy, n = 10 (12.3%)	
Active airway obstruction	8 (80.0%)
Concern for airway obstruction	1 (10.0%)
Oropharyngeal bleeding	1 (10.0%)
Treatment type, n = 81 (100%)	
Antifungal	33 (40.7%)
Antimoniate	29 (35.8%)
Antihelminthic	11 (13.6%)
Antiprotozoal	16 (19.8%)
Antibiotics	7 (8.6%)
Surgery^a^	5 (6.3%)
Treatment outcomes, n = 67 (82.7%)^b^	
Resolution of symptoms	52 (77.6%)
Refractory symptoms	15 (22.4%)

Number and percentage of patients with data available for each category included next to each category header.

^a^ Excludes tracheostomy.

^b^ 11 patients missing outcome data.

Several clinical factors were investigated to assess their relationship with diagnostic accuracy and patient outcomes. Presence versus absence of tracheostomy trended towards but did not achieve significance based on mean time from symptom onset to diagnosis, despite the tracheostomy group having a greater delay in diagnosis (29.4 vs 16.3 months, respectively; *P* = .082). Presence versus absence of immunodeficiency was not associated with a statistically significant difference in the sensitivity of serological test results when considering systemic immunodeficiency alone compared to no immunodeficiency (88.9% positive vs 76.5% positive, respectively; *P* = .402) nor when combining localized and systemic immunodeficiency compared to no immunodeficiency (88.9% vs 77.9%, respectively; *P* = .658).

## Discussion

In this systematic review, we examine diagnostic modalities and factors impacting need for tracheostomy in LL. Leishmaniasis is currently considered endemic in 99 countries and territories, including Latin America, Asia, and Africa.^13^ Increased globalization makes this disease relevant in areas that are historically Leishmania‐free.^14^ The incidence of leishmaniasis in the United States is unknown as it is not a nationally notifiable disease, yet studies have suggested Texas as being a potentially endemic region likely due to its proximity to Mexico.^15^, ^16^, ^17^ In this study, among patients with available data on endemic exposure, 97.3% either presented in, or had reported travel from, a region identified as endemic for leishmaniasis. Nevertheless, 2.7% of patients had no record of exposure to an endemic region, indicating that the absence of endemic exposure cannot be solely relied on to rule out LL. To the best of our knowledge, the incidence and prevalence of LL has not been previously described, making it difficult to compare this study's epidemiological data with that of other studies. However, a recent study does estimate the prevalence of CL and VL between the years 2000 to 2021 to be about 12 million endemic cases compared to about 10,000 nonendemic cases.^3^ The rarity of nonendemic cases can make the decision to pursue a biopsy of laryngeal tissue difficult in patients without endemic exposure. These scenarios introduce the utility of conservative diagnostic modalities, as biopsy may not be an obvious first step when suspicion for LL is low.

Among the available diagnostic modalities for leishmaniasis, biopsy with parasitological analysis has the highest specificity.^18^ The initial concern is often cancer, and thus it is reasonable to pursue a tissue sample when the diagnosis is uncertain. While biopsy is the gold standard for diagnosis of Leishmania, we feel that serology plays a useful role in two clinical scenarios: when there is low suspicion for malignancy and a more conservative approach is preferred, or when biopsy results are inconclusive and additional diagnostic data is needed. In this study, either initial or subsequent biopsy results were positive among 100% of patients who received a biopsy as we only queried the literature for patients with a confirmed diagnosis of LL. However, studies have shown a biopsy sensitivity ranging from 52% to 58% on lymph node biopsy among VL patients,^18^ which may often be the targeted tissue sample in LL patients. Additional studies are needed to determine the sensitivity of biopsy of the larynx in the setting of leishmaniasis. These inconclusive scenarios demonstrate a potential need for additional diagnostic testing.

The most commonly utilized serological tests available for leishmaniasis include IFAT, DAT, rK‐39 ICT and ELISA.^19^ In this study, 44 LL patients (54.3%) received serological testing, with the most commonly used test being IFAT (43.2%). Of these 44 patients, 77.2% had positive serological results, despite all patients in this cohort having a biopsy‐confirmed diagnosis of LL. Studies investigating IFAT for diagnosis of VL in immunocompetent hosts have reported sensitivities ranging from 72% to 100% and specificities from 80% to 100%.^20^, ^21^, ^22^ However, to our knowledge, there is no data available to compare the sensitivity and specificity of serological testing in LL patients to the results of this study. It is notable that only about half of the patients in this study underwent serological testing, despite its known utility in the diagnosis of leishmaniasis. This highlights the need for increased awareness of the diagnostic options in suspected leishmaniasis, specifically in the case of rare manifestations such as LL, where physicians may be hesitant to acquire a biopsy.

Several other diagnostic modalities also exist for leishmaniasis. A recent systematic review with meta‐analysis of American tegumentary leishmaniasis (ATL) found polymerase chain reaction (PCR) to be a feasible means for diagnosis of ATL, with a sensitivity of 95% and specificity of 92% to 97%.^23^ Other commonly employed diagnostic tests for VL include DAT, with a reported pooled sensitivity of 96% (95% CI 92%‐98%) and a pooled specificity of 95% (95% CI 86%‐99%), and rK‐39 ICT, with a pooled sensitivity of 91% (95% CI 88%‐93%) and a pooled specificity of 89% (95% CI 85%‐91%).^24^, ^25^ The diagnostic performance of these tests in LL patients are not available for comparison to the results of this study. Given this sparsity of data in patients with suspected LL, it may be reasonable to approach these cases in a similar manner to the work up of more well known leishmaniasis subtypes, such as VL. Current recommendations for the work up of VL include the simultaneous use of several diagnostic methods, including parasitological examination (eg, biopsy or culture), molecular testing (eg, PCR), and/or serological testing (eg, IFAT or DAT).^26^ Based on a sensitivity of 77.2% across several types of serological tests in this cohort, we posit that serology is a reasonable first step in the work up of patients with suspicion for LL, as positive results can help avoid the need for a biopsy. Among these serological tests, IFAT was most commonly used in this cohort (43.2%), followed by ELISA (20.5%), with sensitivities of 78.9% and 100%, respectively. However, the reliability of these sensitivities is limited due to the inability to compare these results to the available LL literature, as well as due to a small sample size in both the IFAT (n = 19) and ELISA groups (n = 9). Sensitivities for other serological tests included in this study were not calculated due to a limited sample size in these groups. Therefore, practitioners must be aware of the potential for a false negative result, and thus negative serology results should be followed by a biopsy.

In this cohort, isolated LL showed a lower sensitivity on serology compared to systemic leishmaniasis (63.6% vs 90.9%, *P* = .031) (Table 4). Other studies have demonstrated a similar pattern when comparing CL versus VL, as the former is thought to be less reliably diagnosed on serology.^27^ Unfortunately, there is no data on LL for comparison of these results. These findings suggest that serology is not a useful tool for ruling out disease in isolated LL. However, as no prior studies have investigated this, further data is necessary before this can be said with certainty.

Immunocompromise is an important consideration in the clinical assessment and diagnostic work up of leishmaniasis, as it is a well‐established risk factor for development of overt disease.^28^ Both systemic immunosuppression (eg, diabetes mellitus or human immunodeficiency virus), as well as immunosuppression localized to the airway (eg, daily inhaled corticosteroids for asthma treatment) have been linked to the various forms of Leishmania infection, including LL.^29^, ^30^ In addition to the increased risk for development of leishmaniasis, immunosuppression has also been shown to decrease the accuracy of serological tests, as individuals may not harbor antibody levels that are detectable by standard techniques.^31^ Among the 61 patients in this cohort with data on immune status, over half (52.5%) had immunosuppression that was either systemic or localized to the airway. Nevertheless, immunosuppression did not impact the sensitivity of serological tests (77.9% immunocompetent vs 88.9% immunocompromised, *P* = .658). Notably, due to the small sample size for individual diagnostic tests in this cohort, this analysis did not control for the different types of serological tests employed which may confound these results. Also, due to limitations in sample size, immunosuppression type (eg, systemic vs localized) was not controlled for in this analysis. To the best of our knowledge, no other studies report on the accuracy of serological tests in immunosuppressed LL patients. Clinicians should consider this association between immunosuppression and leishmaniasis when stratifying the risk of LL in their patients. Furthermore, the results from this study suggest that clinicians should not be dissuaded from using serological tests in immunosuppressed individuals, as sensitivities were similar when stratified by immune status.

While serological studies are considered a reliable diagnostic modality, several issues with its accuracy in testing for leishmaniasis are recognized in the literature. Most notably, these tests are subject to variable sensitivity based on antigenic differences between Leishmania species, and specificity may be low due to cross‐reactivity with other infectious organisms.^32^ Additionally, the logistics of conducting these tests is made difficult due to the need for sophisticated laboratory equipment for certain serological tests, such as PCR‐ELISA.^33^ Nonendemic regions are particularly susceptible to these logistical issues. Depending on the test used and the availability of resources to process the collected specimens, turnaround time may be lengthy and thus can cause a delay in diagnosis, even when LL is appropriately suspected. For example, the University of Washington served as the primary national reference laboratory for the Center for Disease Control and Prevention from 2021 to 2022. During that time, they reported a median turnaround time of 3.19 days from specimen receipt for molecular diagnosis of leishmaniasis using PCR.^34^ While this turnaround time is reasonable, there is no mention of how long it might take a specimen to arrive at the laboratory after being sent from the original institution. These factors, as well as LL being a difficult diagnosis to suspect in nonendemic regions, are most likely responsible for the delay in diagnosis observed in this study. Lastly, as previously mentioned, antibody tests may be inaccurate in immunosuppressed individuals whose antibody levels may not be detectable by standard techniques (false negatives).^31^ However, this decrease in diagnostic accuracy was not observed in the immunosuppressed patients in this cohort.

The most significant concern with LL is the potential for airway obstruction. In this study, 90.0% of patients who received a tracheostomy either had active airway obstruction or concern for airway obstruction. Notably, the patients in this study had an average of 18.4 months between symptom onset to final diagnosis. It is not uncommon for the diagnosis of LL to be delayed, subsequently resulting in increased morbidity.^35^ While there was a longer delay from symptom onset to diagnosis in patients with a tracheostomy versus those without, this difference was not statistically significant (29.4 vs 16.3 months, respectively; *P* = .082). However, these results may be limited due to a sample size of 10 patients in the tracheostomy group. The greater delay in diagnosis in those who received a tracheostomy may reflect ample time to allow for lesion expansion, leading to airway obstruction. Larger studies assessing the clinical variables correlated with increased morbidity in LL may yield valuable prognosticators for risk stratification in these patients.

Another factor likely contributing to delays in diagnosis is the variability of the clinical presentation of LL. The most common symptoms observed in this cohort included dysphonia (80.2%), dysphagia (21.0%), and odynophagia (14.8%), which may raise suspicion for laryngeal disease, but are all nonspecific. General physical exam findings were similarly nonspecific, such as cutaneous lesions (20.7%), hepatosplenomegaly (15.5%), and lymphadenopathy (6.2%) (Table 1). Although all general physical exam findings were likely due to systemic leishmaniasis and all cutaneous lesions in this cohort were due to active CL or known prior CL, in practice, these lesions may be too nonspecific to raise suspicion for Leishmania in patients without a known history of the disease, particularly in nonendemic areas. Lastly, laryngoscopy findings varied widely and thus lesions were grouped into several general descriptors, including granulomatous (24.2%), erythematous (22.6%), polypoid (22.6%), and several others (Table 2). This variability in clinical presentation and exam findings underscores both the utility of non‐invasive diagnostic testing as well as the need for awareness of LL by the diagnosing physician.

There are several limitations of this study to note. The most notable limitation is related to the rarity of LL, leading to an inherently small sample size in this study. This hindered our ability to perform certain statistical analyses, such as evaluating the performance of certain diagnostic tests in the setting of immunocompromise. The chronology of systemic involvement, if any, in LL patients could not be controlled for in this review given its nature; albeit, the majority of symptoms reported here can be attributed to laryngeal manifestations of leishmaniasis rather than systemic involvement due to the nature of the symptoms. It is important to note that diagnostic accuracy of serologic tests was assessed for all patients with LL irrespective of the presence or absence of concomitant systemic involvement before, during, or after the testing was performed. This may impact serologic accuracy, although this impact has not been previously reported. The studies included in this review were mostly limited to case reports and case series, and only contained English‐language publications, potentially limiting the scope of our data collection and analysis given that leishmania is significantly more common in non‐English speaking countries. Documentation across studies was inconsistent, including incomplete reporting of diagnostic test results and endemic exposure. Studies were also collected across several decades, which may introduce a bias in the work up performed in some patients based on the availability of certain diagnostic tests (eg, rK‐39 ICT was first introduced in the late 1990s).^35^ The specificity of diagnostic tests could not be assessed as all patients in this cohort had a diagnosis of LL, limiting our findings to the sensitivities of these tests only. Due to the inherit selection and information bias in case reports/series, the utility of bias assessments is limited in these study types, which comprise the majority of articles included in this study.

## Conclusion

This study highlights the need for increased awareness of the clinical presentation of LL and the diagnostic options available. This need is demonstrated by a significant delay in the final diagnosis of LL in this cohort. Additionally, despite the well‐documented utility of conservative diagnostic measures in leishmaniasis, only about half of the patients in this cohort underwent serological testing, whereas the majority of patients underwent biopsy. We found that LL most commonly presented in males with reported exposure to a region endemic for leishmaniasis and that immunosuppression did not affect the results of serological studies among LL patients. The most common treatment modalities included antifungal and antimonial drugs which resulted in resolution of symptoms in most. Requirement for tracheostomy was not statistically associated with a delay in diagnosis, but this finding may be limited by a small sample size.

## Author Contributions

**Joseph Celidonio**, data acquisition, statistical analysis, drafting, revision, final approval; **Nicholas Hamilton**, data acquisition, drafting, revision, final approval; **Alexandra Filipowski**, data acquisition, drafting, revision, final approval; **Raj Malhotra**, drafting, revision, final approval; **Samantha Shave**, drafting, revision, final approval; **Myat Mon**, data acquisition, revision, final approval; K**enneth Yan**, design, analysis, final approval; **Rachel Kaye**, conception, design, analysis, final approval.

## Disclosures

### Competing interests

None.

### Funding source

None.

## Supporting information


**Supplemental Figure 1.** A) Numerous spherical to ovoid‐shaped intracellular amastigotes of leishmaniasis measuring 1‐5 μm long by 1‐2 μm wide. B) Small spheroid intracytoplasmic organisms, amastigotes located within macrophages of the sebaceous gland, consistent with Leishmania.

**Supplemental Figure 2.** A‐C) Post‐treatment nonspecific epiglottic edema in a patient with laryngeal leishmaniasis.

Supporting File 1.
