# Supplementary material for: Clinical and Diagnostic Considerations in Laryngeal Leishmaniasis: A Systematic Review
Source: OTO Open. 2026 Jul 30;10(3):e70281. doi: 10.1002/oto2.70281 (PMC13421088; doi:10.1002/oto2.70281)
Supplement: Supplementary file 1 — Supplemental Figure 1. A) Numerous spherical to ovoid‐shaped intracellular amastigotes of leishmaniasis measuring 1‐5 μm long by 1‐2 μm wide. B) Small spheroid intracytoplasmic organisms, amastigotes located within macrophages of the sebaceous gland, consistent with Leishmania. [file OTO2-10-e70281-s002.pdf]

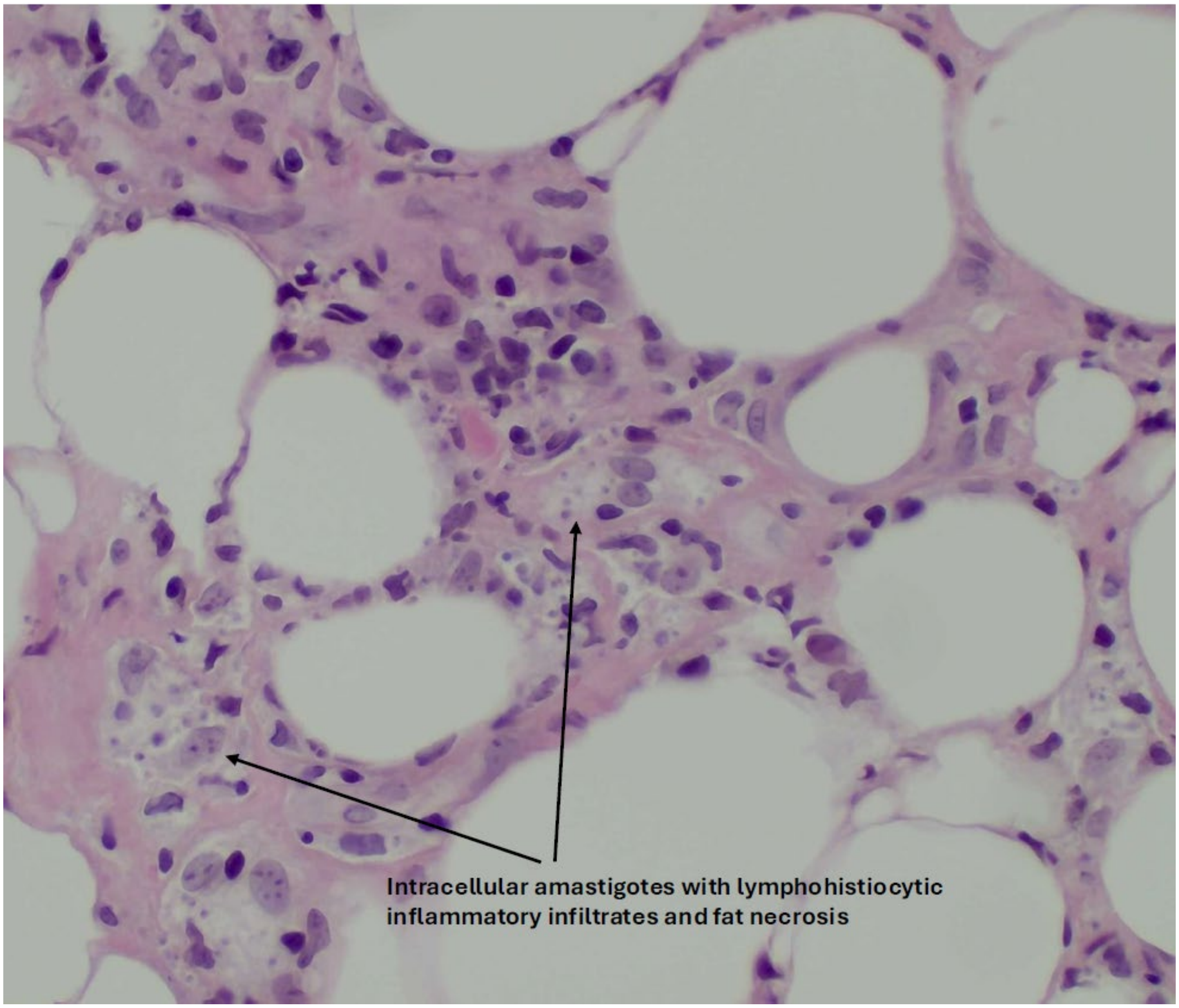
Intracellular amastigotes with lymphohistiocytic
inflammatory infiltrates and fat necrosis

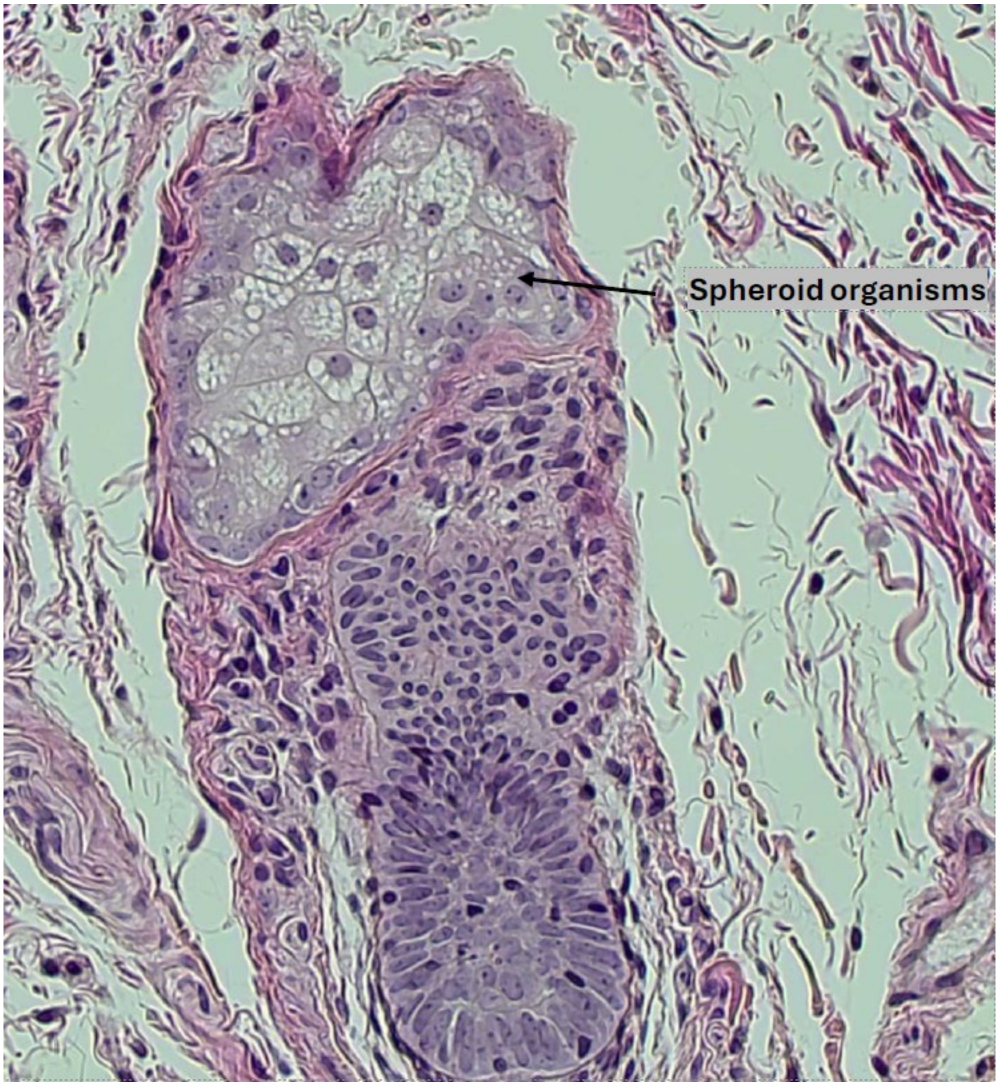
Spheroid organisms
