# Supplementary figures and images for: Clinical and Diagnostic Considerations in Laryngeal Leishmaniasis: A Systematic Review
Source: OTO Open. 2026 Jul 30;10(3):e70281. doi: 10.1002/oto2.70281 (PMC13421088; doi:10.1002/oto2.70281)

A

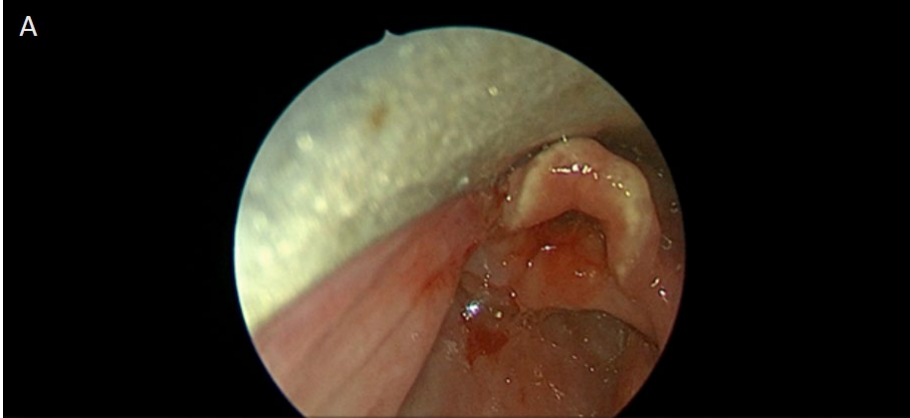

B

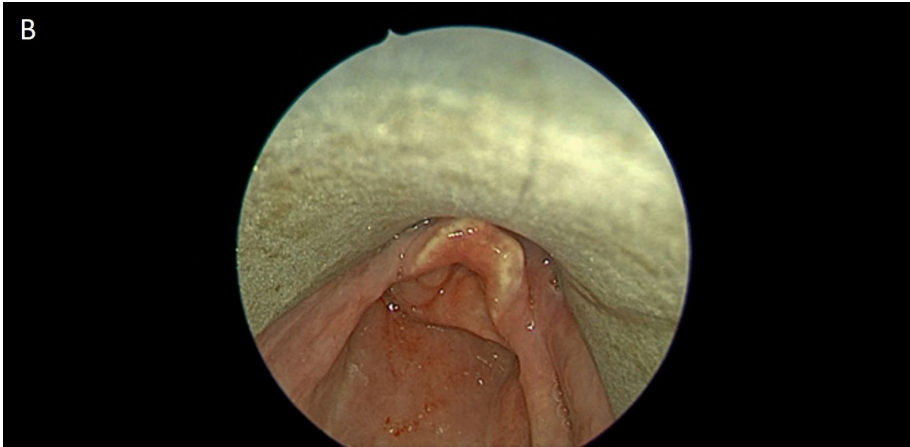

C

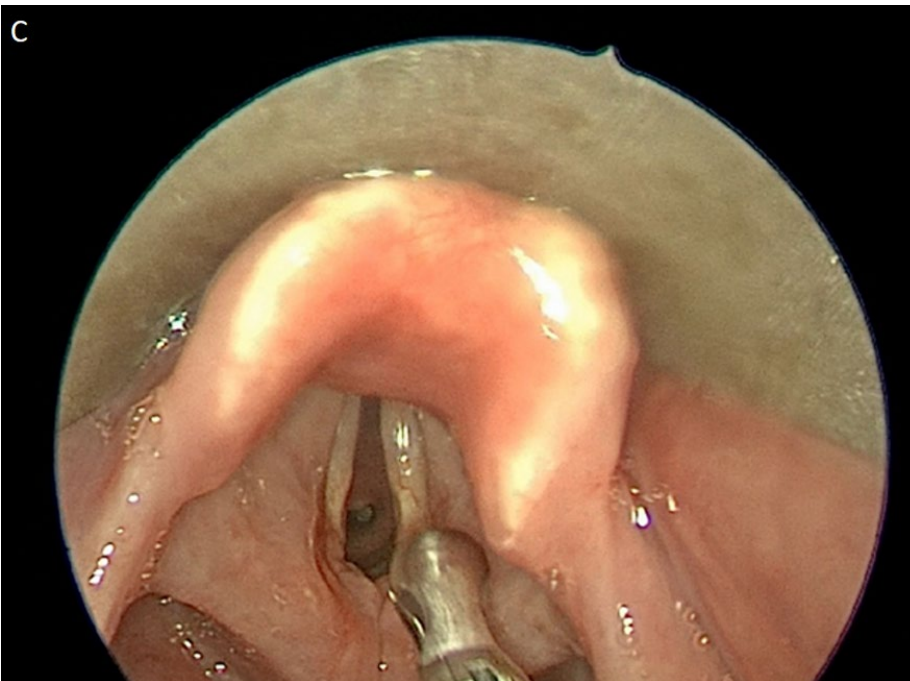

Supplement: Supplementary file 2 — Supplemental Figure 2. A‐C) Post‐treatment nonspecific epiglottic edema in a patient with laryngeal leishmaniasis. [file OTO2-10-e70281-s001.pdf]
