# Supplementary material for: Clinical and Diagnostic Considerations in Laryngeal Leishmaniasis: A Systematic Review
Source: OTO Open. 2026 Jul 30;10(3):e70281. doi: 10.1002/oto2.70281 (PMC13421088; doi:10.1002/oto2.70281)
Supplement: Supplementary file 3 — Supporting File 1. [file OTO2-10-e70281-s003.docx]

**Supplemental Table 1.** Bias Assessment of Included Articles

| **Study** | **Selection Bias** | **Performance Bias** | **Attrition Bias** | **Detection Bias** | **Reporting Bias** |
| --- | --- | --- | --- | --- | --- |
| Moraes et al. | N/A - case report | No bias | No bias | No bias | No bias |
| Aggarwal et al | N/A - case report | No bias | No bias | No bias | No bias |
| Renard et al. | N/A - case report | No bias | No bias | No bias | No bias |
| Grant et al. | N/A - case report | No bias | No bias | No bias | No bias |
| Patel et al. | N/A - case series | No bias | No bias | No bias | No bias |
| Marsden et al. | N/A - case report | No bias | No bias | No bias | No bias |
| Costa et al | N/A - case report | No bias | No bias | No bias | No bias |
| Jha et al. | N/A - case report | No bias | No bias | No bias | No bias |
| Iqbal et al. | N/A - case report | No bias | Lacks follow-up data | No bias | Lacks outcome data |
| Tiseo et al. | N/A - case report | No bias | No bias | No bias | No bias |
| Cocuzza et al. | N/A - case report | No bias | Lacks follow-up data | No bias | No bias |
| Fsadni et al | N/A - case report | No bias | No bias | No bias | No bias |
| Lopes et al. | N/A - case report | No bias | Lacks follow-up data | No bias | No bias |
| Santangeli et al. | N/A - case report | No bias | Lacks follow-up data | No bias | No bias |
| Roberts et al | N/A - case report | No bias | No bias | No bias | No bias |
| Lazzeroni et al. | N/A - case report | No bias | Lacks follow-up data | No bias | No bias |
| Teemul et al. | N/A - case report | No bias | Lacks follow-up data | No bias | No bias |
| Bajraktari et al. | N/A - case report | No bias | No bias | No bias | No bias |
| Silva et al. | N/A - case report | No bias | No bias | No bias | No bias |
| Lella et al. | N/A - case report | No bias | No bias | No bias | No bias |
| Kumar et al. | N/A - case report | No bias | No bias | No bias | No bias |
| Fernandez-Flores et al. | N/A - case report | No bias | Lacks follow-up data | No bias | No bias |
| Basile et al. | N/A - case report | No bias | No bias | No bias | No bias |
| Shirian et al. | N/A - case series | No bias | Lacks follow-up data | No bias | Lacks outcome data |
| Navarro et al. | N/A - case report | No bias | No bias | No bias | No bias |
| Souza de et al | N/A - case report | No bias | Lacks follow-up data | No bias | No bias |
| Gokmen et al. | N/A - case report | No bias | Lacks follow-up data | No bias | No bias |
| Calvopina et al. | N/A - case report | No bias | No bias | No bias | No bias |
| Canovas et al. | N/A - case report | No bias | Lacks follow-up data | No bias | No bias |
| Cobo et al | N/A - case series | No bias | Lacks follow-up data | No bias | No bias |
| Guddo et al. | N/A - case report | No bias | Lacks follow-up data | No bias | No bias |
| Granel et al. | N/A - case report | No bias | No bias | No bias | No bias |
| Patel et al. | N/A - case series | No bias | No bias | No bias | No bias |
| Casolari et al. | N/A - case report | No bias | No bias | No bias | No bias |
| Cunchillos et al. | N/A - case report | No bias | No bias | No bias | No bias |
| Nandy et al. | N/A - case report | No bias | Lacks follow-up data | No bias | Lacks outcome data |
| Vazquez-Pineiro et al. | N/A - case report | No bias | No bias | No bias | No bias |
| Zan et al. | N/A - case report | No bias | Lacks follow-up data | No bias | No bias |
| Terceiro et al. | N/A - case report | No bias | No bias | No bias | No bias |
| Campbell et al. | N/A - case report | No bias | No bias | No bias | No bias |
| Sbroglio et al. | N/A - case report | No bias | No bias | No bias | No bias |
| Drabe et al. | N/A - case report | No bias | No bias | No bias | No bias |
| Fisicaro et al. | N/A - case report | No bias | Lacks follow-up data | No bias | No bias |
| Orlando et al. | N/A - case report | No bias | No bias | No bias | No bias |
| Badaro et al. | N/A - case series | No bias | No bias | No bias | No bias |
| Zinneman et al. | N/A - case report | No bias | Lacks follow-up data | No bias | No bias |
| Flor et al. | N/A - case report | No bias | Lacks follow-up data | No bias | Lacks outcome data |
| Adu-Gyamfi et al. | N/A - case report | No bias | No bias | No bias | No bias |
| Santos et al. | N/A - case report | No bias | No bias | No bias | No bias |
| Sevestre et al. | N/A - case report | No bias | No bias | No bias | No bias |
| Motta et al. | No bias | No bias | No bias | No bias | No bias |
| Marsden et al. | N/A - case series | No bias | Lacks follow-up data | No bias | Lacks outcome data |
| Faucher et al. | No bias | No bias | Lacks follow-up data | No bias | Lacks outcome data |
| Gonzalez-Anglada et al. | N/A – case series | No bias | No bias | No bias | No bias |
| Aliaga et al | N/A - case series | No bias | Lacks follow-up data | No bias | No bias |

Selection Bias: Does the design or analysis account for important confounding and modifying variables through matching, stratification, multivariable analysis, or other approaches?; Performance Bias: Did researchers rule out any impact from a concurrent intervention or an unintended exposure that might bias results?; Attrition Bias: If attrition was a concern, were missing data handled appropriately (e.g., intention-to-treat analysis and imputation)?; Detection Bias: Were interventions/exposures assessed/defined using valid and reliable measures, implemented consistently across all study participants?; Reporting Bias: Were the potential outcomes prespecified by the researchers? Are all prespecified outcomes reported?
